# Supplementary material for: Graphene-Based Far-Infrared Therapy Promotes Adipose Tissue Thermogenesis and UCP1 Activation to Combat Obesity in Mice
Source: Int J Mol Sci. 2025 Feb 28;26(5):2225. doi: 10.3390/ijms26052225 (PMC11900916; doi:10.3390/ijms26052225)
Supplement: Supplementary file 1 [file ijms-26-02225-s001.zip › ijms-3470367-supplementary.pdf]

Supporting Information

Supplementary Figure

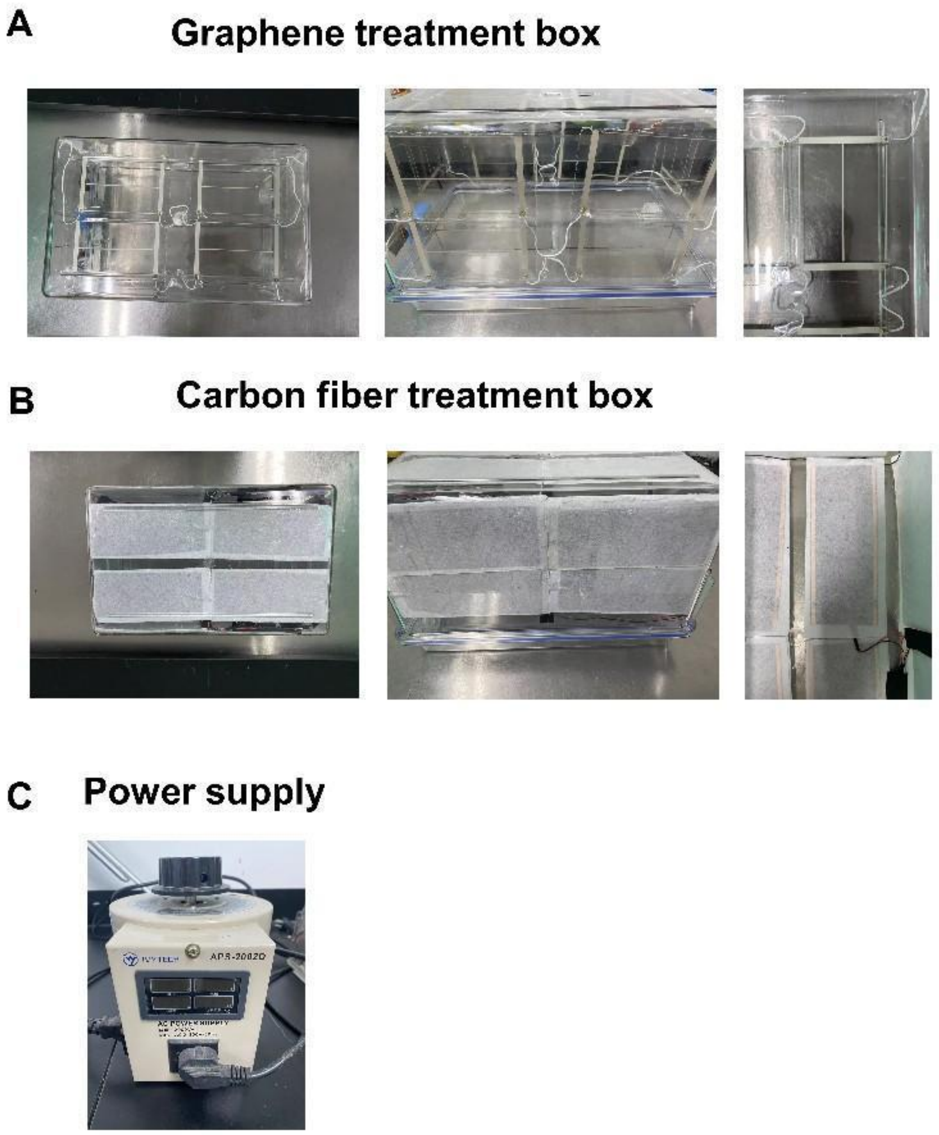

Supplementary figure S1. Photos of FIR radiation device (A) Photograph of a graphene film. (B) Photograph of a carbon fiber film. (C) Power supply.

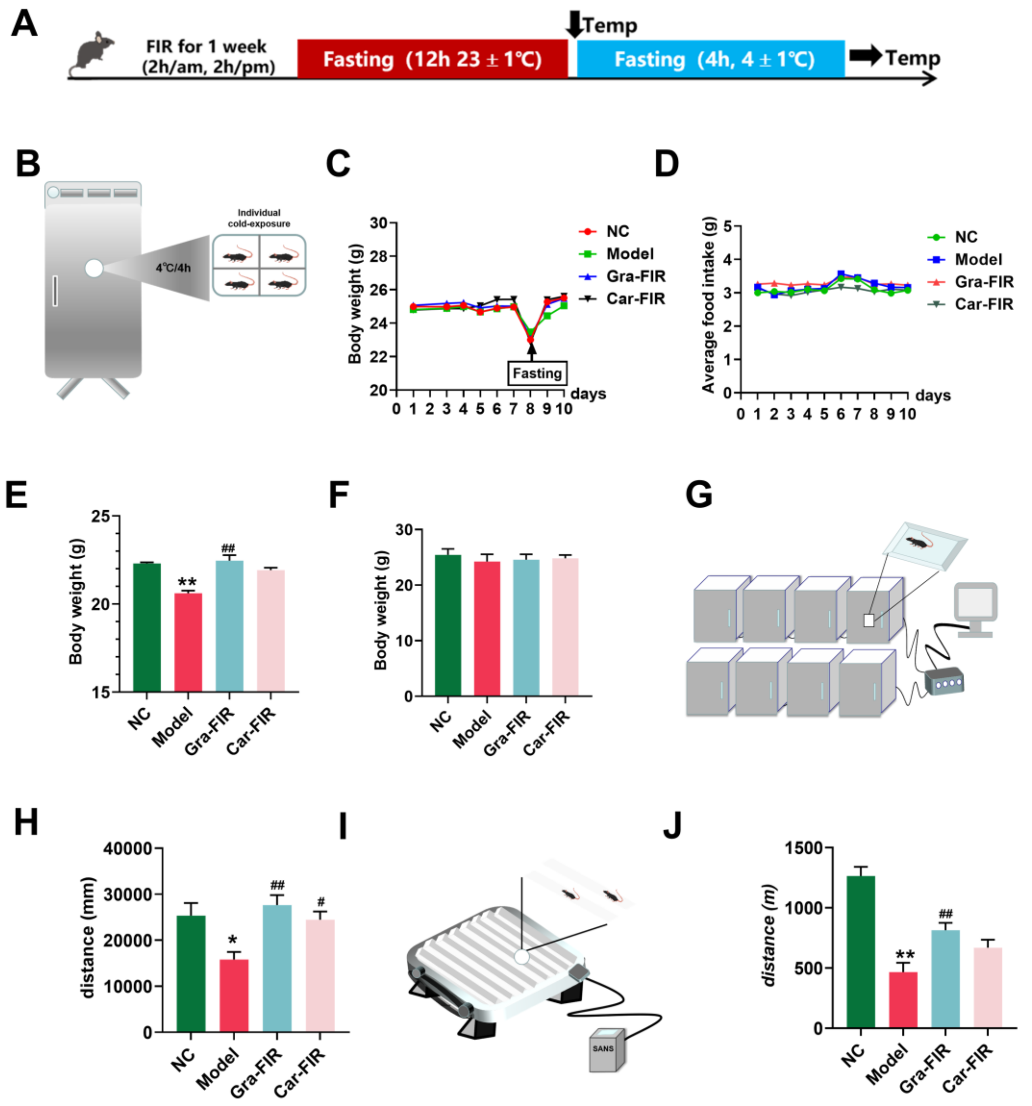

Supplementary figure S2. Effect of graphene-FIR therapy on the phenotype of cold-exposed mice

(A) Experimental procedure; (B) Schematic of cold exposure; (C) Line chart of body weight of mice in each group; (D) Line chart of food intake of mice in each group; (E) body weight of mice after the first cold exposure; (F) body weight of mice after final cold exposure; (G-H) spontaneous activity diagram and data statistics diagram of mice in each group; (I-J) Schematic diagram of running platform an-d data statistics chart of mice in each group. The statistical data is displayed as mean±SEM; n = 8-10, **P*<0.05, ***P*<0.01 VS NC group; $^{\#}P$<0.05, $^{\#\#}P$<0.01 VS Model group.

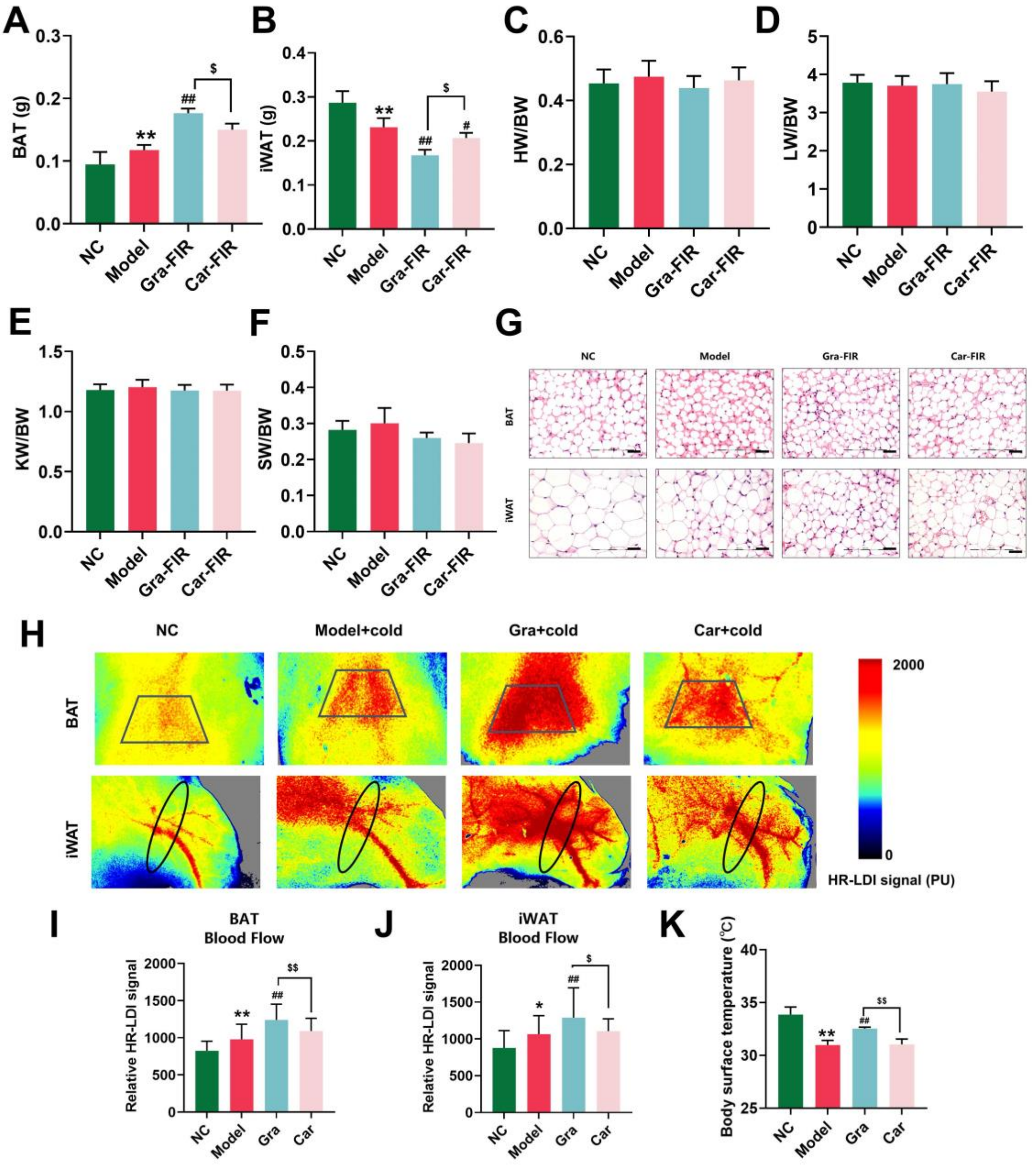

Supplementary figure S3. Graphene-FIR therapy on the influence of adipose tissue in mice to cold exposure

(A-B) The weight of BAT and iWAT in each group; (C-F) Organ indexes of heart, liver, kidney and spleen in each group; (G) HE staining of BAT and iWAT of mice in each group (200X), Scale-bar=50 μm; (H-J) groups of BAT in mice and the amount of blood flow perfusion iWAT schematic diagram and data statistics; (K) Statistical analysis of body surface temperature of mice in each group. The statistical data is displayed as mean±SEM; n = 8-10, *P<0.05, **P<0.01 VS NC group; $^{\#}P$<0.05, $^{\#\#}P$<0.01 VS Model group.

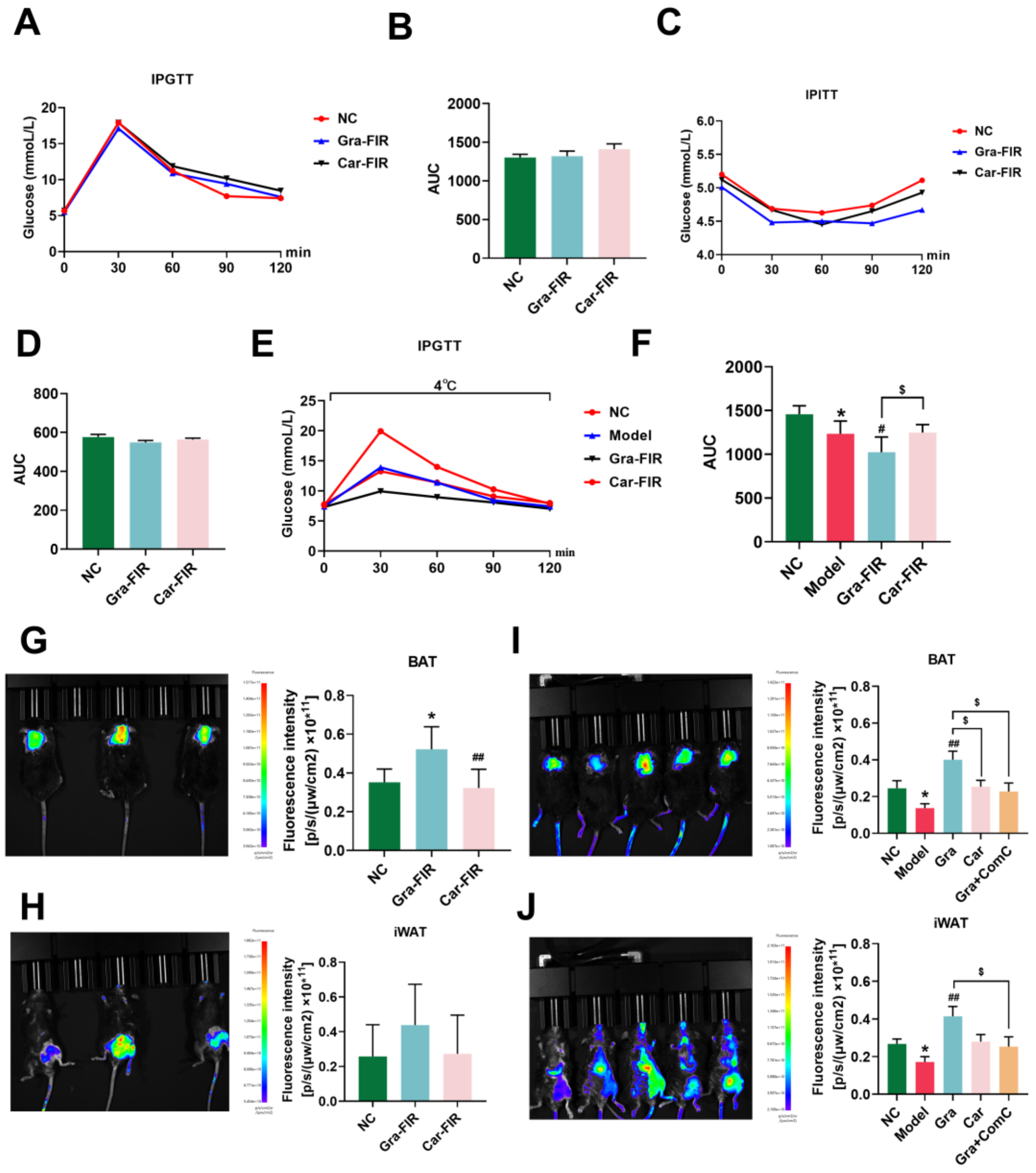

Supplementary figure S4. Graphene-FIR therapy enhanced cold exposure of glucose tolerance in mice and adipose tissue glucose uptake

(A-B) IPGTT and AUC of each group after FIR therapy; (C-D) IPITT and AUC of each group after FIR therapy; (E-F) IPGTT and AUC of each group after cold exposure; (G-H) Representative images and statistical analysis of glucose uptake in adipose tissue of each group of mice after FIR therapy; (I-J) Representative pictures of glucose uptake in adipose tissue of mice in each group after cold exposure and statistical analysis. The statistical data is displayed as mean ± SEM; n = 8-10, *P<0.05, **P<0.01 VS NC group; $^{\#}P$<0.05, $^{\#\#}P$<0.01 VS Model group.

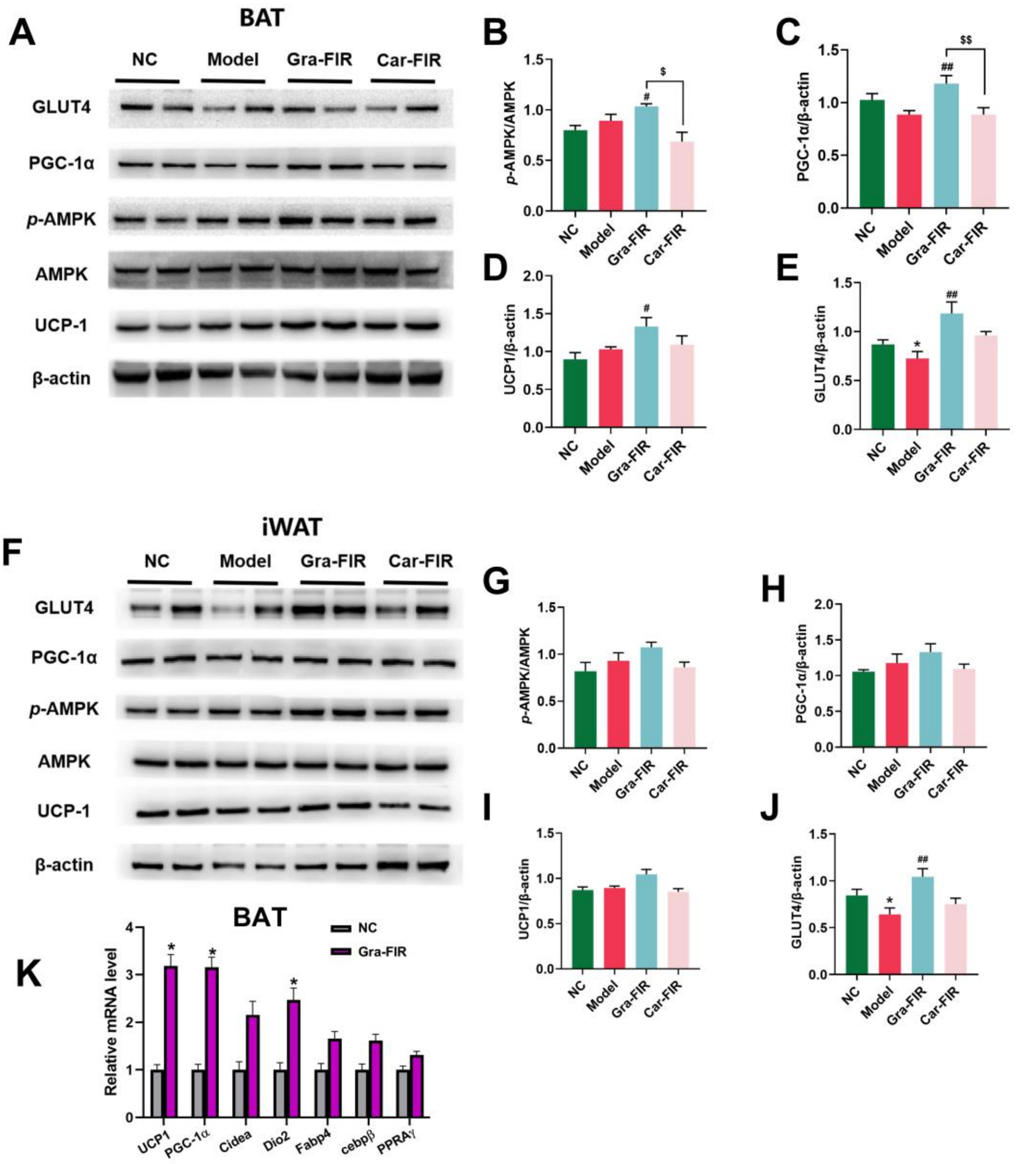

Supplementary figure S5. Graphene-FIR therapy enhanced the increase in thermogenesis of BAT in cold-exposed mice

(A-E) Representative protein bands and relative protein expressions of AMPK, PGC-1α, UCP1, and GLUT4 in BAT. β-actin was used as a loading control. (F-J) Representative protein bands and relative protein expressions of AMPK, PGC-1α, UCP1, and GLUT4 in BAT. (K) The mRNA expression of thermogenic genes. The statistical data is displayed as mean±SEM; n = 6, *P<0.05, **P<0.01 VS NC group; $^{\#}P$<0.05, $^{\#\#}P$<0.01 VS Model group.

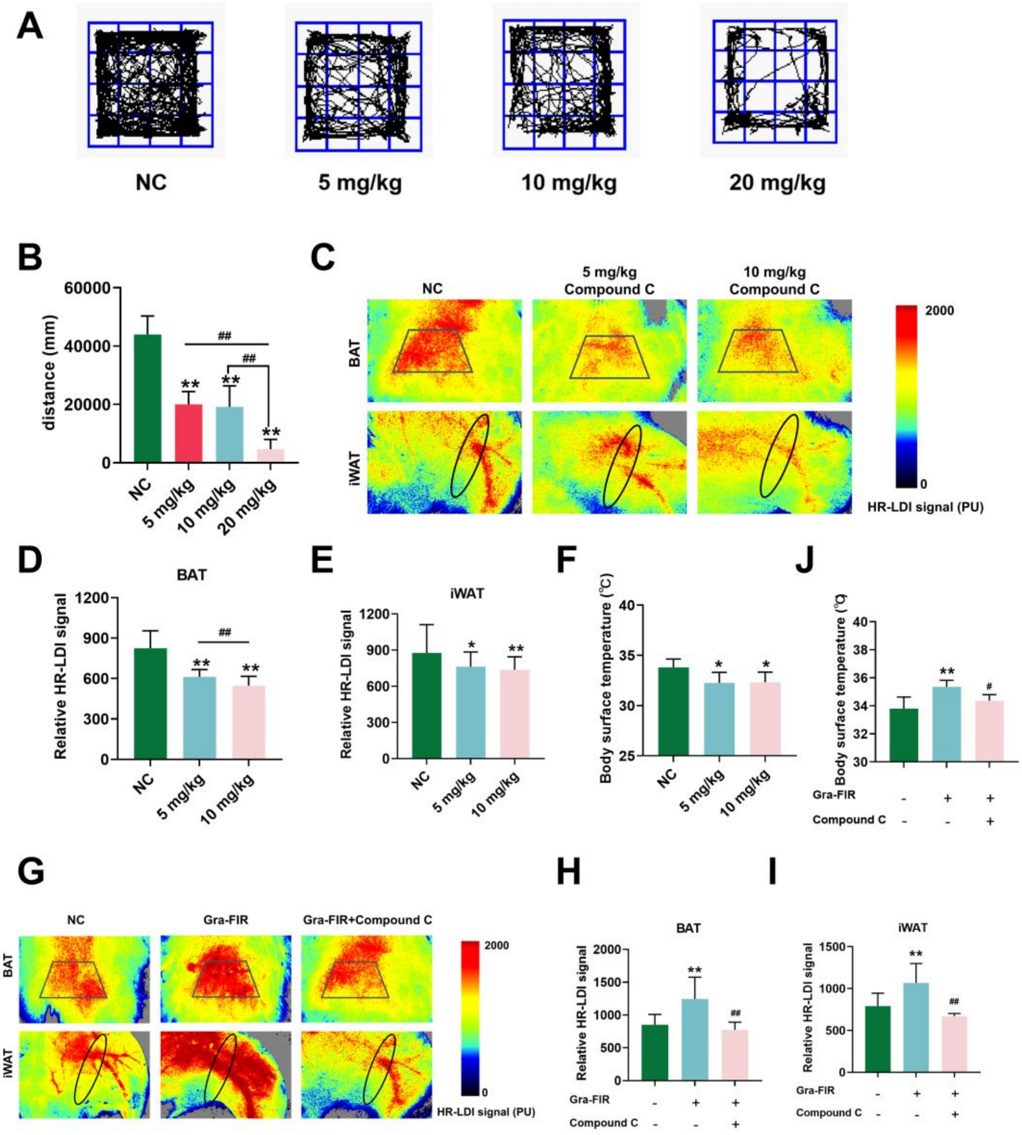

Supplementary figure S6. Effect of AMPK inhibitor on adipose tissue blood flow and glucose uptake in mice with graphene-FIR therapy

(A-B) Locomotor activity of AMPK inhibitor in mice; (C-E) Schematic diagram and data statistics of blood perfusion in BAT and iWAT of mice induced by AMPK inhibitor; (F) effect of AMPK inhibitor on body surface temperature; (G-I) Schematic diagram and statistical diagram of AMPK inhibitor on the blood perfusion of BAT and iWAT in mice after Gra-FIR therapy; (J) Effect of AMPK inhibitor on body surface temperature of mice after Gra-FIR therapy. The statistical data is displayed as mean ± SEM; n = 6, *P<0.05, **P<0.01 VS NC group; $^{\#}P$<0.05, $^{\#\#}P$<0.01 VS Gra-FIR group.

Supplementary materials and methods

2.1. Materials

Decolorization shaker (Haimen Qilin Beier Instrument Manufacturing Co., LTD.); Electronic balance (Sartorious, Germany); Mill-Q ultra-pure water system (Millipore, USA); Protein electrophoresis device and power supply (Beijing Wix Technology Co., LTD.); Ultra-sensitive automatic imaging analyzer (Protein Simple Company, USA); Freezing grinding instrument (Beijing Hede Technology Co., LTD., model: Beijing N9548R); Victor3 automatic microplate reader (Molecular DeviceS, USA); Frozen high speed centrifuge (eppendorf, Germany, Model: 5424R).Graphene and carbon fiber heating sheets were provided by the Grahope New Materials Technologies Inc (Shenzhen, China). CY5.5-Glucose dye was purchased from Qiyue Biology (Xian China) dissolved in 5% DMSO. Dorsomorphin (Compound C, 5% DMSO solution, cat:HY-13418A, MCE. USA). Anti-P-AMPK (Thr172) (1:1000, #2235S, CST, USA), Anti-AMPKα (1:1000, #2532S, CST, USA), anti-GLUT4 (1:1000, #2213S, CST, USA); anti-UCP1 (1:5000, ab10983, Abcam, UK), anti-PGC-1α (1:500, sc-518025, Santa Cruz, China), anti-β-actin antibody (1:1000, Zenbio, China); Horseradish peroxidase labeled goat anti-mouse IgG (1:5000,ZB2305) and horseradish peroxidase labeled goat anti-rabbit IgG (1:5000, ZB2301) were purchased from Beijing Zhongshan Jinqiao Biotechnology Co., LTD. ECL luminescent solution and PVDF membrane were purchased from Milipore Company; The BCA kit (AR1097, Boster, Wuhan, China). Mouse insulin Kit (CSB-E05071m, Cuasbio, Wuhan, China), mouse Leptin kit (JL11317, Jianglaibio, Shanghai, China), mouse Adiponectin kit (JL20696, Jianglaibio, Shanghai, China).

2.2. Infrared spectrum characterization and analysis

The infrared spectrum was collected using a Fourier transform infrared spectrometer (Thermo Fisher, Nicolet iS50). The room temperature was kept at 25 °C. The deuterated triglycine sulfate was selected for the detector, and KBr was used for the beam splitter. The final spectrum was recorded by wavelength. An attenuated total reflectance external fiber optic probe assembly with Ge probe material was connected

to the Fourier infrared spectrometer equipment through a coupler. Put the probe in the air to collect background data first and then kept close to the surface of the FIR fiber for collect data. The obtained data were processed by advanced ATR correction and the calculation formula is

$$\text{out} = (\text{in} - \min(\text{in}))/(\max(\text{in}) - \min(\text{in}))$$

In and out are the input and output vectors, respectively, and min and max return the minimum and maximum values, respectively, of the input vector.

The similarity between the different spectra was evaluated by Pearson correlation coefficients (PCCs) and Euclidean distance. The PCC is a statistical indicator that measures the correlation between two variables; it is defined as the quotient of the covariance and standard deviation with two variables. The PCC is defined as follows:

$$\rho_{x,y} = \frac{\mathrm{cov}(x,y)}{\sigma_x \sigma_y} = \frac{\mathrm{E}[(x-\mu_x)(y-y_x)]}{\sigma_x \sigma_y}$$

Euclidean distance, also called the Euclidean metric, is a commonly used distance definition that refers to the true distance between two points in m-dimensional space. The Euclidean distance is defined as follows:

$$d(A,B) = \sqrt{(a_1-b_1)^2 + (a_2-b_2)^2 + \dots + (a_n-b_n)^2}$$

The smaller the Euclidean distance between the vectors, the greater the similarity. The similarity between vectors A and B is defined as follows:

$$\mathrm{E}_{A,B} = \frac{1}{1+\mathrm{d}(\mathrm{A},\mathrm{B})}$$

2.3 Feed composition and energy proportion

Nutrient composition table of NC group feed and high-fat diet feed with a total weight of 25 kg per bag of feed. The proportion of protein, carbohydrate and fat content of the feed is determined by the company at the time of production, The production conditions were in accordance with the provisions of GB 14924.1-2001 "General Quality Standard of compound Feed for Laboratory Animals", GB 14924.2-2001 "Hygienic Standard of Compound Feed for Laboratory Animals", and GB14924.3-2010 "Nutritional Composition of Compound Feed for Laboratory Animals". The energy conversion coefficient of the feed was determined by the feed

production company, and the energy value of the NC diet was 3.52 kcal/g (SPF-F02-001, Biotechnology Co, Ltd. Beijing, China), and the energy value of the high-fat diet was 5.24 kcal/g (D12492, Biotechnology Co, Ltd. Beijing, China). According to the reference value of nutrition, the specific calculation method is as follows: energy conversion coefficient (kcal/g) = protein (g) ×4.0 kcal + carbohydrate (g) ×4.0 kcal + fat (g) ×9.0 kcal.

The energy intake was calculated by equation based on food intake (MF) and energy conversion coefficient (Re = 3.52 or 5.24 kcal/g for NC chow or HFD chow).

Energy intake = Re × MF

Table S1 Proportion of dietary energy in mice

	Ratio of mass (g%)		Power feed ratio (kcal%)	
Project	NC group	High-fed diet	NC group	High-fed diet
Protein	19.16	26	21.7	20
Carbohydrates	57.74	26	65.5	20
Fat	5.0	35	12.8	60
Energy value			3.52 kcal/g	5.24 kcal/g

Table S2 The main nutritional test values of NC group diet (Item No. 1010013)

Main components	Test results	Reference range
Water content	96	≤100
Crude protein	212.4	≥200
Crude fat	56	≥40
Crude fiber	35	≤50
Crude ash	60	≤80
calcium	11.5	10-18
Total phosphorus	7	6-12
Aflatoxin B1	Not detected	≤20.0
Total colonies	<10	≤5×104

Table S3 The main nutritional test values of High-fat diet feed (Item No. D12492)

Main components	Content of ingredients (%)
Casein protein	25.845
Cystine	0.388
Maltodextrin	16.153
Sucrose	8.891
Cellulose	6.461
Soybean oil	3.231
Lard	31.660
Multiple minerals M1002	1.292
Calcium hydrogen phosphate	1.680
Calcium carbonate	0.711
Potassium citrate	2.132
Multivitamins 1001	1.292
Choline tartrate	0.258
Eat blue dye	0.005

2.4 FIR irradiation intensity calculation

The formula for FIR irradiation intensity is as follows:

$$\eta = S\sigma\ (Tr4\text{-}T04) \times 100/Pe.$$

η represents the irradiation intensity and S represents the heating area (where the heating area of monolithic graphene is 120 mm × 80 mm = 9600 mm2), the heating area of a single piece of carbon fiber is 160 mm × 70 mm = 11200 mm2), σ stands for Stefan-Boltzmann constant, σ = 5.67×10-8 W/(m2·K4), Tr is the average radiation temperature in Kelvin absolute temperature scale (K), T0 is the ambient temperature in the Kelvin absolute temperature scale (K), and Pe represents the measured electrical power in watts (W). According to the formula, we can obtain that the irradiation intensity of the graphene-FIR device is higher than that of the carbon fiber-FIR device. Our results showed that the heating of graphene is stable and high

electrothermal transform efficiency.

2.5 HE staining

The fixed liver and adipose tissues were taken and sectioned in paraffin, followed by deparaffinization, staining, dehydration and sealing. The morphological changes of liver tissue, adipose tissue (white adipose tissue and brown adipose tissue) of mice in each group were observed under a light microscope.

HE staining detailed steps: 1) Deparaffinization: paraffin sections were put into 10 min in xylene I-10 min in xylene II-5 min in absolute ethanol I-5 min in absolute ethanol II-5 min in 95% alcohol 5 min-90% alcohol 5 min-80% alcohol 5 min-70% alcohol 5 min-distilled water and washed; 2) Hematoxylin staining: the sections were stained with hematoxylin for 3 min, washed with tap water for 15 min, differentiated in 1% hydrochloric acid alcohol for several seconds, washed with tap water, returned to blue in 0.6% ammonia water, and rinsed with running water. 3) eosin staining: sliced into eosin staining fluid dyeing in 3 min. 4) Dehydration sealing: The slices were placed in 95% alcohol I for 5 min-95% alcohol II for 5 min-absolute alcohol I for 5 min-absolute alcohol II for 5 min to be dehydrated, and then placed in xylene I for 5 min-xylene II for 5 min to be transparent. The slices were taken out of xylene and dried, and sealed with neutral gum. 5) take pictures: observed under optical microscope (Nikon, Japan) histopathological change form.

2.6 Oil red O staining

The mouse liver tissues fixed with 4% formaldehyde were prepared in liquid nitrogen environment to make frozen sections with a thickness of 10μm. The sections were placed on slides coated with polylysine and fixed in 10% formaldehyde fixative for 10 minutes. The slices were washed slightly with distilled water, and then dried. The slices were stained in oil red working solution for 8-10 minutes. The color was separated by 60% ethanol. Slightly washed with distilled water; Hematoxylin was used to stain the nuclei for 30s. Tap water is blue; Dry the surrounding water with filter paper, seal the piece with glycerin gelatin; The content of lipid droplets in liver tissue was observed under light microscope.

2.7 RNA extraction and quantitative real-time PCR

1) Total RNA Extraction: Total RNA was extracted from tissue samples by Total RNA Extraction Kit (DNase I) (GenePool, Cat: GPQ1801). 2) RNA electrophoresis: 5μl of RNA was subjected to electrophoresis on 1% agarose gel to check the integrity of RNA. 3) reverse transcription: after using mRNA cDNA short Kit (GenePool and Cat# GPQ1803) reverse transcription, experiments according to product manuals. 4) Real Time PCR in BIOER LineGene 9600 plus type fluorescent quantitative PCR, the $2\text{-}^{\triangle\triangle CT}$ method carries on the data of relative quantitative analysis. Primer-related information is shown in Table S1.

Table S4 Primer sequences used for semi-quantitative RT-PCR analysis

Gene	Primer	5'-3'sequence
PPARγ	Forward	CTCCACACTATGAAGACATTCCAT
	Reverse	CCACAGACTCGGCACTCAA
PGC-1α	Forward	TACGCACAACTCAGCAAGTC
	Reverse	CCAGTCACAGGAGGCATCTT
Cidea	Forward	CTGACAAGAGCCACCAACATC
	Reverse	TGCGAGCAGCGAATAGTTCT
Dio2	Forward	GGACTCCTCAGCGTAGACTTG
	Reverse	CAGGAAGAGGCAGTTGGAGAA
Prdm16	Forward	ATATGCGAGGTCTGCCACAAG
	Reverse	GGAGGAGGTAGTGCTGAACATC
Elovl3	Forward	ATTACATCTGGAGGCAGGAGAA
	Reverse	TAGGCTCGGTGGAAGAAGTG
β-actin	Forward	TGGGTATGGAATCCTGTGGC
	Reverse	ATGCCTGGGTACATGGTGG

2.8 Western blot

After 50 mg adipose tissue (BAT, iWAT) were added to the lysate, ground and allowed to stand on ice for 30 min, the supernatant was extracted by centrifugation at 12,000 rpm for 20 min at 4℃. Total protein concentrations in adipose and colon

tissues were determined using the BCA Protein assay kit. The later supernatant was mixed with loading buffer, denatured at 100℃ for 15 min, and stored at -80℃ until use. Twenty μg of total protein was wet transferred to PVDF membrane by SDS-PAGE. Seal with 5% skim milk for 2 h at room temperature, wash with TBST 3 times (5 min each time), Then *P*-AMPK (1:1000), AMPK (1:1000), β-actin (1:5000), PGC-1α (1:500), UCP1 (1:5000), SIRT1 (1:1000) were incubated with different primary antibodies and incubated overnight at 4℃ in a shaker. After that, the cells were washed three times with TBST for 5 min each time. The bands were incubated in the corresponding secondary antibody (1:5000) for 1 h at room temperature. After that, the bands were washed three times by TBST, and the developer solution was uniformly covered on the bands. Bands were visualized using the Protein Simple imaging system (FluorChem E 5.0.4, FE1159, USA)and analyzed using Image J software (National Institutes of Health, USA).

2.9 Exercise endurance test

Exerise endurance was determined using a treadmill (Sansbio, Jiangsu, China) running tests as previously described with minor modifed. Te low and high-intensity exercise test were defned by setting diferent running time.

Prior to exhaustion running, mice were pre-adapted to the treadmill for 10 min per day for 3 days at a gradually increased speed (2 to 10 m/min), training is necessary to ensure that mice are familiar with the treadmill and task. On the training day, mice allow to freely explore the treadmill for 1-3 min and slowly increase the speed from 2m/min to 10m/min. If the mice could not finish the training process, it will be excluded. On the test day, set the electric shock intensity appropriately 2.55 mA and treadmill protocol: 14m/mim(2min)→16m/mim(3min)→18m/mim(25min)→20m/mim(15min)→22m/mim(15min)→24m/mim(15min)→26m/mim(15min)→28m/mim(90min). If mouse stay in the fatigue zone, be prepared to intervene by tapping the mouse with a wire brush or tail tickling. If a mouse remains in the fatigue zone for 5 continuous sec, promptly remove the mouse from the treadmill and record the duration and distance it ran.

2.10 Locomotor activity

To examine the efect of different doses of AMPK inhibitors on the locomotor activity, after the last AMPK inhibitors (Intraperitoneal Administration,), locomotor activity was examined in a 15 min monitoring period. Te movement tracking was recording by using anautomated video tracking system, as described previously. Eight test boxes (40 cm×40 cm×30 cm high) were operated simultaneously by a compatible computer.

2.11 Surface body temperature

Te surface temperature of subjects (mice and FIR flm) was tested by the infrared imaging. IR imaging tested in a room with a steady temperature and humidity. Termographic camera (N18198648, Wuhan Yoseen Infrared Co., Ltd., China) was recorded temperature in mice or FIR flm.

2.12 Blood fow measurements/laser speckle contrast imaging (LSCI)

To prevent the carryover efects of difering acclimation temperatures, all mice were placed at 25 °C for 1 h before to research. Te mice were placed in an RFLSI Pro (RWD life sciences) and kept at 1.2% isofurane. A CCD camera detects blood fow and then captures the image. Regions of interest (ROIs) were chosen and assess speckle contrast.

2.13 Fluorescence imaging analysis

In vivo fuorescence imaging was detected and quantifed by AniView600 imaging system (Guangzhou Biolight Biotechnology Co., Ltd). To reduce autofuorescence, animals were fasted for 12 h and hair was removed from joints. Animals underwent caudal vein injection solution of Cy5.5-glucose (2.5 mg/kg). As recommended by the system for image acquisition, the excitation and emission flters were set at 675 nm/Cy5.5 and 740 nm/Cy5.5, respectively. The mice were scanned and observed within half an hour after FIR irradiation or cold exposure. We calculated average Cy5.5- glucose signal in BAT and iWAT.

2.14 Hyperthermia therapy in human subjects

Ten healthy individuals (10 males) with a mean age of 35 years and a normal BMI were included in the study. Subjects were instructed to follow a similar daily schedule, including meal timing and composition, rest/activity time, and amount of

exercise, and to avoid strenuous exercise, caffeine, drug, or alcohol intake in the week prior to the experiment to avoid circadian/lifestyle effects on thermogenesis. For FIR hyperthermia, each subject was in a relaxed state in a familiar temperature and humidity controlled room, where they sat in an upright position with arms folded, wore a standard test gown, and their head, neck, and shoulders were unclothed to ensure visualization of clavicle surface temperature. The test chamber was kept quiet and strictly undisturbed, and all volunteers were asked to maintain a relaxed position throughout the test with minimal movement to avoid unwanted effects on body heat.

Subjects were acclimated in the test chamber 1 h before baseline imaging. After baseline imaging, participants' supraclavicular fat stores (around the upper shoulder/upper back region) were exposed to a graphene-FIR phototherapy chamber for 45 minutes, during which thermal imaging photographs were taken.Subjects had thermal images of their supraclavicular fat depots taken 20 min after stopping FIR treatment. After that, the exact same experimental protocol was repeated for each volunteer.
